# Inversion of Electromagnetic Models for Bare Soil Parameter Estimation from Multifrequency Polarimetric SAR Data

**DOI:** 10.3390/s8128181

**Published:** 2008-12-11

**Authors:** Nazzareno Pierdicca, Paolo Castracane, Luca Pulvirenti

**Affiliations:** Department of Electronic Engineering, Sapienza University of Rome, via Eudossiana 18, 00184 Rome Italy; E-mails: pierdicca@die.uniroma1.it, pulvirenti@die.uniroma1.it

**Keywords:** Radar polarimetry, SAR, bare soil, soil moisture

## Abstract

The potentiality of polarimetric SAR data for the estimation of bare soil geophysical parameters (i.e., roughness and soil moisture) is investigated in this work. For this purpose, two forward models available in the literature, able to simulate the measurements of a multifrequency radar polarimeter, have been implemented for use within an inversion scheme. A multiplicative noise has been considered in the multidimensional space of the elements of the polarimetric Covariance Matrix, by adopting a complex Wishart distribution to account for speckle effects. An additive error has been also introduced on the simulated measurements to account for calibration and model errors. Maximum a Posteriori Probability and Minimum Variance criteria have been considered to perform the inversion. As for the algorithms to implement the criteria, simple optimization/integration procedures have been used. A Neural Network approach has been adopted as well. A correlation between the roughness parameters has been also supposed in the simulation as a priori information, to evaluate its effect on the estimation accuracy. The methods have been tested on simulated data to compare their performances as function of number of looks, incidence angles and frequency bands, thus identifying the best radar configuration in terms of estimation accuracy. Polarimetric measurements acquired during MAC Europe and SIR-C campaigns, over selected bare soil fields, have been also used as validation data.

## 1. Introduction

The sensitivity of bare soil radar backscattering to soil moisture content and roughness has been demonstrated by several studies, both experimental and theoretical (e.g., [1-2]). However, the difficulty to separate the contribution of the various surface characteristics (both dielectric and geometric) influencing the radar signal, the ill-position of the forward problem (i.e., different combinations of surface properties may give rise to the same backscattering coefficient *σ^0^*), and the large amount of unknown effects on the radar measurements, make the retrieval of bare soil parameters from microwave radar data a challenging problem. To tackle these difficulties, several pieces of information should generally be introduced in the retrieval process, such as prior information about the quantities to be retrieved [3-7]. The presence of vegetation further complicates the situation, even though an attempt to estimate soil moisture over vegetated areas has been recently carried out [8].

Discriminating the contribution of soil moisture and surface roughness to the backscattered radar signal is a crucial aspect when dealing with the retrieval problem [9]. If a single polarization and single frequency synthetic aperture radar (SAR) system is used, bare soil multi-parameter estimation is an ill-posed problem since one measurement is used to estimate more than one unknown. If the objective is the retrieval of one target parameter, the others are assumed to be nuisance ones (see also [10], in which a bistatic radar configuration is investigated), and their effect should be minimized both by choosing an appropriate radar configuration (for instance, observation at low incidence angle, if moisture is the target parameter) and introducing *a priori* information [7]. To overcome these limitations, radar multifrequency and multipolarization data can be used, including SAR polarimetric observations. The multidimensional information obtained helps separating roughness and moisture effects.

Different approaches have been adopted to deal with the retrieval problem both from single- and multiparameter SAR data. Generally, empirical/semi-empirical techniques and physical methods are distinguished [7, 11]. The former are based on experiments providing large datasets matching radar measurements and geophysical data. By fitting in some way the experimental data, they can provide a relationship yielding the radiative quantity as a function of the instrument parameters (frequency, observation angle, polarization) and of the target condition, i.e., the forward model given as a “model function”. Alternatively, an inverse relation to directly retrieve the geophysical parameters can be derived [11-13]. Backscattering coefficients can be taken as such in the inverse relation or they can be combined in a non-linear way (i.e., ratio between polarizations) to compensate as much as possible the influence of undesired effects. A main drawback is the difficulty in characterizing the whole range of ground truth parameters, i.e., training the algorithm on a representative experimental database without limiting the range of applicability.

Theoretical electromagnetic models physically describe the soil electromagnetic properties and the scattering mechanisms [14, 15]. They simulate the radar measurement in the presence of specific characteristics of the terrain, usually represented in terms of material (i.e., dielectric) and geometrical (i.e., roughness) properties, and as a function of the sensor parameters. Physical models have the possibility to deal with a large number of situations [16]. However, the forward model is not necessarily expressed in a closed mathematical form, so that it becomes unfeasible to find out a closed form solution for inverting it. Moreover, considerable discrepancies between simulations and actual radar data may occur, thus preventing the possibility of reliably estimating soil parameters by model inversion [11]. In other words, uncertainties in the forward model cause retrieval inaccuracies.

Despite the differences between retrieval approaches discussed above, the same statistical criteria should drive the retrieval process. Regression techniques are very often used to solve the problem, especially in the framework of empirical approaches when some linear relation between predictors and parameters to be retrieved can be always postulated [17]. The use of non linear functions of the basic radar measurements can overcome the difficulties represented by the non-linearity of the forward process. Neural Networks have been extensively used to invert models [16, 18-20]. The role of multiparameter data to be combined in non linear way (i.e., ratio between backscattering coefficients) has been put in evidence in this context [18]. The Kalman technique has also been tested showing its high flexibility [21]. The same flexibility is a main advantage of techniques based on Bayesian theory of parameter estimation, which has been considered by several authors for processing SAR images. Besides the extensive use of Bayesian techniques to analyze, classify or restore SAR images [22-24], their roles in soil parameter retrieval have been also consolidated [16, 25, 26].

The objective of this work is to investigate the potential of estimating bare soil parameters (soil moisture *m_v_*, roughness standard deviation *s* and correlation length *l*) using SAR polarimetric data. In addition, an evaluation of the most suitable sensor configuration for retrieving bare soil geophysical quantities is provided. The problem of inverting forward models (either empirical or theoretical) is stated within the Bayesian theory of parameter estimation, following the same approach used for classifying SAR polarimetric images in [22]. We have accounted for the speckle noise that has been considered in the multidimensional space of the elements of the polarimetric Covariance Matrix. Different inversion schemes have been compared. We have distinguished between criteria to be followed for estimating the parameters and algorithms able to implement the criteria. Maximum a posteriori probability and minimum variance criteria have been implemented by a Monte Carlo minimization/integration approach. A Neural Network approach has been used as well. To identify the best sensor configuration, the comparison has been performed using different sets of radar parameters.

We have applied the retrieval algorithms to both simulated and real data. The Integral Equation Model (IEM) [27-29] and a Semiempirical Model (SEM) [12, 30] have been implemented to simulate polarimetric SAR measurements. The synthetic dataset has been generated for single and multilook data. An additive error has been also introduced to evaluate the effect of calibration and model errors on the retrieval accuracy. The consideration of prior information on the parameters has been introduced in this work by assuming the existence of a linear correlation between *s* and *l*, and the improvement of the retrieval accuracy due to this assumption has been evaluated.

The analysis of the simulated data has allowed us to identify the best system parameters (frequency, polarization and incidence angle) to estimate soil moisture and roughness. The experimental part of the work makes use of measured data available from airborne MAC Europe and from spaceborne SIR-C campaigns, both over the Italian test site of Montespertoli, Florence. Some bare soil fields have been selected in the images, whose roughness was determined by different rural tillage (ploughed, harrowed and rolled fields). The retrieval algorithms have been applied to the polarimetric radar signatures of the fields, where corresponding ground truth measurements were available for comparison.

The comparison of different estimation approaches, as well as the evaluation of the best sensor configuration, aims at yielding a contribution to find a reliable road to solve the problem of bare soil parameter retrieval from radar data. However, because of the numerous complications discussed at the beginning of this section, an ultimate solution to this problem is still far from being obtained.

The work is organized as follows. Section 2 provides a brief review on polarimetric radar data and on the direct models used to simulate them. Section 3 describes the methodologies we have used to estimate soil parameters, i.e, both the criteria followed for estimating the parameters and the algorithms implementing the criteria. Section 4 discusses the results and Section 5 reports the concluding remarks.

## 2. Forward models for simulating polarimetric SAR data

### 2.1. Polarimetric SAR data

A polarimetric radar is able to measure the 2×2 Scattering Matrix **S** that relates the electric field scattered by each pixel of the radar image to the incidence electric field. **S** is therefore formed by the complex (both amplitude and phase) backscattered signals in four combinations of the linear received and transmitted polarizations: *hh*, *hv*, *vh*, and *vv* (where *h* and *v* stand for horizontal and vertical polarizations, respectively). Since reciprocity applies to natural targets, the radar information is retained in only three elements of **S**. This information can be represented by a 3-element complex vector **x**:

(1) $$X=\left[\begin{array}{c} S_{\text{hh}}\\ S_{\text{hv}}\\ S_{\text{vv}} \end{array}\right]$$

where **x** follows a zero mean multivariate Gaussian distribution [22], [31] and *s_hv_*=*s_vh_* (assuming reciprocity).

SAR data are usually multilook processed for speckle reduction. In this case, the Mueller matrix, which relates the scattered modified Stokes vector to the incidence Stokes vector, is generally introduced. It is well known that, instead of the Mueller matrix, it is possible to refer to the so called polarimetric Covariance Matrix **C**:

(2) $$C=\langle XX^{*T}\rangle =\left[\begin{array}{ccc} \langle S_{\text{hh}}S_{\text{hh}}^{*}\rangle & \langle S_{\text{hh}}S_{\text{hv}}^{*}\rangle & \langle S_{\text{hh}}S_{\text{vv}}^{*}\rangle \\ \langle S_{\text{hv}}S_{\text{hh}}^{*}\rangle & \langle S_{\text{hv}}S_{\text{hv}}^{*}\rangle & \langle S_{\text{hv}}S_{\text{vv}}^{*}\rangle \\ \langle S_{\text{vv}}S_{\text{hh}}^{*}\rangle & \langle S_{\text{vv}}S_{\text{hv}}^{*}\rangle & \langle S_{\text{vv}}S_{\text{vv}}^{*}\rangle \end{array}\right]$$

where <·> denotes ensemble averaging and superscripts * and *T* indicate complex conjugation and transposition, respectively.

A multilook radar yields a maximum likelihood estimator of **C** [22], [31], that is, it provides matrix **Z** as in the following:

(3) $$Z=\frac{1}{n}\sum_{k=1}^{n}x(k)x{\left(k\right)}^{*T}$$

where index *k* identifies each individual measurement sample and *n* is the number of looks. When the radar resolution cell is made by many elementary scatterers, the matrix **A**=*n***Z** has a complex Wishart distribution [22]. This means that the probability density function (pdf) of **A** given a target with Covariance Matrix **C**, that is the conditioned pdf *p*(**A**|**C**), is given by:

(4) $$p(A|C)=\frac{{\left|A\right|}^{n-q}\exp[-\text{Tr}(C^{-1}A)]}{K(n,q){\left|C\right|}^{n}}$$

where |·| indicates the determinant, *Tr*(·) represents the trace operator, *q*=3 is the dimension of vector **x** and *K*(*n*,*q*) is a function whose expression can be found in [22, 31]. The Covariance Matrix **C** depends on incidence angle and frequency, so that a multiangle and multifrequency radar system supplies several matrices **Z**(*t*), where index *t*=1:*T* indicates the *t*-th measurement configuration (whose total number is *T*). Note that, in case a single polarization radar is considered (measuring, for instance, only the backscattering coefficient in *hh* polarization: σ^0^*_hh_*=4πs_hh_s_hh_*), Equation (4) reduces to the χ^2^ distribution of order 2*n*, as discussed in [1]. The latter is more realistic than the Gaussian pdf to describe radar observations affected by speckle noise.

A forward model maps the three soil moisture and roughness parameters, i.e., *m_v_*, *s*, *l*, into the Covariance Matrix ***C***=***C***(*m_v_*, *s*, *l*). The measured **Z** differs from **C** for several reasons, such as the instrumental errors, the residual speckle after look summation, and the influence of further soil parameters affecting the radar response, that have not been accounted for in the forward model. Inhomogeneities within the radar resolution cell may also represent a source of discrepancy since the forward relation ***C***(*m_v_*, *s*, *l*) is generally non-linear.

### 2.2. The adopted literature forward models

A very fundamental aspect in the development of rough surface scattering models is the way the randomness of the surface is characterized. In this paper, we refer to the classic description of the roughness as a stationary bivariate random process, which is described by the autocorrelation function or, alternatively, by the power density spectrum. An exponential autocorrelation function has been adopted. The vertical scale of the roughness is described by the standard deviation *s*, while the horizontal scale of the roughness is described by the correlation length *l* [1].

The first direct model considered here has been proposed by Oh *et al.* and it is based on a semi-empirical (SEM) approach [12]. In successive works the authors extended this approach to the case of a fully polarimetric radar, that is, they have also considered the cross-product <s_hh_s_vv_*> between *hh* and *vv* polarized returns [32, 33]. More recently, the model has been further updated [30], and it now exhibits a dependence to surface correlation length, although quite small. The model is semi-empirical since it has been developed on the basis of electromagnetic considerations deriving from the theory of the Small Perturbation Method, and of the Kirchoff Method (both for Geometric Optics and Physical Optics approximations) to make assumptions about the functional relations between soil parameters and some of the terms of **C**. Experimental results and best fitting techniques have been used to derive the actual values of numerical parameters appearing in the model itself and/or expressions for other terms of **C** (such as amplitude and phase of <s_hh_s_vv_*>).

The second direct model is referred to in the literature as the Integral Equation Model (IEM), since it is a closed form solution of the integral equation applying to the surface electromagnetic field at the boundary between the air and a rough soil. The solution has been proposed by Fung *et al.* and subsequently refined [27-29, 34-36]. Here we have adopted the version named IEMM, and the transition from the incident angle to normal angle for the Fresnel reflection coefficient (see related references) is done as proposed in Fung textbook, adding an interpolation to avoid abrupt discontinuities. The model consists of iterating the Kirchoff approximation of the surface field in the integral equation in order to find out a more accurate solution. A fairly manageable (but still quite complicated) solution is derived by considering the far region approximation for the observed field, that is, an observation point far enough from the scattering portion of the surface. By introducing further approximations, an expression of **C** is derived. It includes different terms in the form of series of integrals whose mathematical expression can be found in the relevant references. For numerical computations the number of needed terms of the series is related to the characteristics of the surface and the wavelength. Some of these terms consider single scattering mechanisms, while other terms account for multiple scattering, relevant for very rough surfaces.

An exponential autocorrelation function (ACF) has been adopted in this work. The choice of the correct ACF is still object of discussion and it seems that the exponential ACF is better for small roughness, whilst the Gaussian one better predicts rough surface scattering. As for the soil permittivity, a model proposed in the literature that relates it to soil moisture and composition on a wide frequency spectrum has been used [37, 38].

## 3. Retrieval methodology

**Figure 1.**
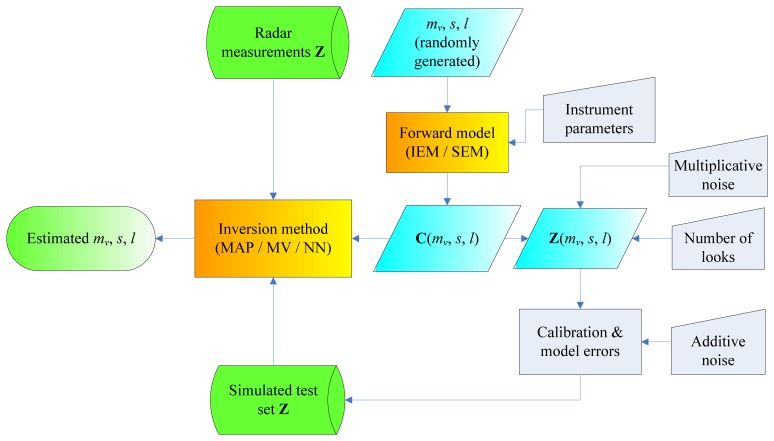
Logical scheme of the procedure adopted in this work.

The logical scheme of the procedure adopted in this work is shown in Figure 1. An electromagnetic forward model is able to describe, at a certain level of accuracy, the interaction of the radar pulse with the rough soil and predicts a radiative quantity (i.e., the backscattering coefficient *σ^0^* or the entire covariance matrix **C**) associated to the soil condition. This depends on soil and system parameters. The radar observations may differ from such expected radiative quantity and therefore the retrieval technique should be able to overcome this difficulty. In this work, a synthetic database {**Z**; *m_v_*, *s*, *l*} combining radar observations and corresponding soil parameters has been generated using forward and error models (the simulated test set in Figure 1) to be used as a surrogate of the radar measurements to perform the retrieval in the first part of the paper.

The inversion method should be able to infer the soil parameters from the measurements. This requires knowledge about the direct mapping ***C***(*m_v_*, *s*, *l*). Here, this kind of information is yielded by the two direct models introduced in the previous section. To build the database, the soil parameters have been generated randomly with uniform distribution within predefined ranges. We paid attention not to overcome too much the limit of validity of the models in our simulations, but at the same time to consider a range for the parameters as wide as possible. The maximum surface slope standard deviation we have considered is 0.5, whilst the range of products between wavenumber and roughness standard deviation (*ks*) and wavenumber and correlation length (*kl*) were: *kl*=12-41/7-23/1.6-5, *ks*=0.2-6/0.1-3.4/0.03-0.8 at X/C/L band. They should be compared with model validity range, such as those reported in [30] and [39].

From the computed covariance matrices **C**(*m_v_*, *s*, *l*), the measured matrices **Z** have been simulated considering different sources of discrepancy between **Z** and **C**. The generation of statistical realizations of measured **A**, given a target whose covariance matrix is **C**, has been performed starting from a Gaussian number generator with zero mean and variance equal to 0.5. The technique is described in [22]. It is worth mentioning that the simulated set only applies when the multilook is obtained by summing samples of the same radar target composed by a large number of scatterers, i.e., a “fully developed” speckle, and the homogeneity of the target within the averaging sample set has been assumed. Besides conventional summation in the frequency domain to generate multilook images, the multilook process can correspond also to subsequent low pass filtering in the spatial domain, or simply to pixel summation within a homogeneous region (“per field” analysis).

In order to take into account further sources of discrepancies (i.e., calibration errors, model errors, etc.), an additive error has been also introduced in the simulation, as described in a subsequent paragraph.

Whilst soil moisture *m_v_* can be assumed to be an independent parameter, roughness standard deviation *s* and correlation length *l* could be, in principle, considered statistically related. For instance, it has been shown that they are not intrinsic properties of natural surfaces, but they may depend on the length and resolution of the roughness profiles collected on ground [6, 40], presenting a positive correlation for short profile length. Moreover, in [41], a sort of calibration of *l*, expressed by a deterministic relationship with *s*, is proposed to adjust the IEM model for better reproducing actual data (i.e., an “equivalent” *l* is introduced). These considerations have suggested us to develop a retrieval strategy that may include a priori information about a statistical dependence between *s* and *l*. Therefore, an additional synthetic data set has assumed *m_v_* and *s* uniformly distributed, while *l* has been randomly generated imposing a linear correlation with *s*. It is left to the reader the decision to exploit such prior information in its retrieval procedure.

### 3.1. The Bayesian approach

The Bayesian theory of parameter estimation has been considered to infer *m_v_*, *s*, *l*, which form a 3-element parameters vector **Θ**. The approach is directly derived from the work proposed by Lee *et al.* in the framework of the classification of polarimetric images [22], and adopts a complex Wishart statistical distribution for the polarimetric measurements. To our knowledge, this is a distinctive aspect that the paper introduces in the framework of soil moisture retrieval, compared to other statistical assumptions, like for instance Gaussian distributions, less suitable to describe radar observations. By using the Bayes theorem, given the measured matrix **Z**, the pdf *p*(**Θ**|**Z**) of a certain soil parameter vector **Θ** conditioned to **Z** is:

(5) $$p(\Theta |Z)=\frac{p(Z|\Theta )p(\Theta )}{p(Z)}=\frac{p(Z|C_{\Theta })p(\Theta )}{p(Z)}$$

where the pdf *p*(**Z**|**Θ**) of measurement **Z** conditioned to **Θ** equals the pdf of **Z** conditioned to the Covariance Matrix **C_Θ_** that is univocally related to the soil parameters of the considered radar target. An estimation rule that maximizes the posterior probability *p*(**Θ**|**Z**) (maximum posterior probability estimator: MAP) can be derived by computing the natural logarithm of equation (5). Using the Wishart distribution for *p*(**A**|**C_Θ_**) [see equation (4)] and considering that **A**=*n***Z**, the MAP criteria reduces to minimize the following distance (or “discriminant function”) with respect to the parameter vector **Θ** [22]:

(6) $$d(\text{Z};\Theta )=n[\ln(\left|C_{\Theta }\right|)+\text{Tr}(C_{\Theta }^{-1}Z)-\ln[p(\Theta )]$$

where we have considered that, whenever matrix **A** is provided, *p*(**A**) is a constant and does not influence the minimization. The same applies to terms *K*(*n,q*) and |**A**|*^n−q^*. In case there is not any a priori information about the probability of the soil parameter *p*(**Θ**)=*p*(*m_v_*, *s*, *l*), the criterion reduces to a Maximum Likelihood (ML) estimator and the distance to be minimized does not depend on the number of looks *n*.

If, instead of searching for the maximum *p*(**Θ**|**A**), we compute the expected value of this distribution, we obtain a minimum variance (MV) estimator that minimizes the root mean square (rms) error (also called minimum mean square estimator). By imposing that the integral of *p*(**Θ**|**Z**) is normalized to 1 and using the Bayes formula, we can develop a criterion to estimate a given element *θ*(*j*) of the vector of the parameters **Θ** that consists in computing the following quantity:

(7) $$\overset{⌢}{\theta }(j)=\int_{D}\theta (j)p(\Theta |Z)d\Theta =\frac{\int_{D}\theta (j)p(Z|\Theta )p(\Theta )d\Theta }{\int_{D}p(Z|\Theta )p(\Theta )d\Theta }$$

*D* is the three-dimensional domain in which the soil parameter a priori pdf is greater than zero and we have considered that *p*(**Z**,**Θ**)= *p*(**Z**|**Θ**) *p*(**Θ**). By substituting the expression of *p*(**Z**|C_**Θ**_) given by the Wishart distribution in Equation (7), with simple manipulations we obtain:

(8) $$\overset{⌢}{\theta }(j)=\frac{\int_{D}\theta (j)\exp[-d(Z;\Theta )]d\Theta }{\int_{D}\exp[-d(Z;\Theta )]d\Theta }$$

where *d*(**Z**;**Θ**) is given by equation (6), so that the argument of the exponential in equation (8) is a function equal to the MAP distance multiplied by −1. Both criteria, MAP and MV, require to compute *d*(**Z**;**Θ**) first, and than search its maximum or evaluate the integral (8).

In the case of *T* statistically independent measurements **Z**(*t*) (e.g., different radar configurations), the joint pdf *p* [**Z**(1),**Z**(2),…,**Z**(*P*)|**Θ**] equals the product *p* [**Z**(1)|**Θ**]·*p* [**Z**(2)|**Θ**]…*p*** [Z**(*P*)|**Θ] ** [42]. The discriminant function becomes simply the summation over *T* of each discriminant function *d* [**Z**(*p*);**Θ**] as in the following equation, and the same applies to the exponent appearing in the MV criterion:

(9) $$d[Z(1),Z(2),\ldots ,Z(P);\Theta ]=\sum_{t=1}^{T}n(t)\{\ln(\left|C_{\Theta }(t)\right|)+\text{Tr}[C_{\Theta }{\left(t\right)}^{-1}Z(t)]\}-\ln[p(\Theta )]$$

In the absence of prior information on the soil parameters, apart from their ranges of variability, they are assumed statistically independent with uniform distribution, so that *p*(**Θ**) is given by:

(10) $$p(\Theta )=p(m_{v},s,l)=p(m_{v})p(s)p(l)=\Pi_{\Delta m_{v}}(m_{v}-{\bar{m}}_{v})\Pi_{\Delta s}(s-\bar{s})\Pi_{\Delta l}(l-\bar{l})$$

being Π_Δ_*_θ_*(*θ*) a rectangular function of width Δ*θ*, and *θ̅* the mean value of the parameter. A hypothetical correlation among *s* and *l* discussed before can be managed by imposing:

(11) $$p(\Theta )=p(m_{v})p(s,l)=p(m_{v})p(s)p(l|s)=\Pi_{\Delta m_{v}}(m_{v}-{\bar{m}}_{v})\Pi_{\Delta s}(s-\bar{s})\Pi_{\Delta l}(l-\alpha s-\beta )$$

where *α* and *β* and Δ*l* determine the range of variability of *l* as well as its correlation coefficient with *s*. In this case, Δ*l* is the spreading of the uniform conditioned pdf *p*(*l*|*s*), while the correlation coefficient between *l* and *s* is $\Delta s/\sqrt{\Delta s^{2}+{\left(\Delta l/\alpha \right)}^{2}}$.

In order to compute MAP and MV estimates, minimization and integration techniques have to be implemented, respectively. We have used a simple method based on a Monte Carlo approach. It requires the statistical generation of a database {**C**; *m_v_*, *s*, *l*}, with soil parameters {*m_v_*, *s*, *l*} randomly generated (with uniform distribution, in this case) and **C** derived from the forward model, as previously discussed. Given a measured **Z** (either a simulated or a real one), the discriminant function is computed for each sample of the database (each realization of the randomly generated soils), that is *d*(***Z***; *m_v_*, *s*, *l*) is sampled randomly within the *D* domain. The MAP criterion is implemented by searching the realization in the {**C**; *m_v_*, *s*, *l*} database that yields the minimum *d* (**Z**; *m_v_*, *s*, *l*). The MV estimate is derived by using a Monte Carlo integration for which the integral of any function *f*(**g**) can be approximated by the arithmetic mean of *f*(**g**)/*p*(**g**) over *H* available samples, where *p*(**g**) is the statistical distribution of **g** [43]. Note that *H* (i.e., the number of records of the database) has to be chosen in order to ensure that both the minimization and the integration are accurate enough. In the following computation, we have generated a database of 10,000 elements.

### 3.2. The Neural Network approach

As mentioned, Neural Network (NN) approaches have been widely used for soil parameter retrieval from radar data (e.g., [16], [18-20]). An artificial NN is a nonlinear parameterized mapping from an input vector **u** to an output vector **v**=NN(**u**; **ω**, *M*), where **ω** is the weight vector (including the biases as well) and *M* is the architectural model of the network. It is well known that a multilayer feedforward neural network, having at least one hidden layer, can approximate any nonlinear function relating inputs to outputs [44].

A feed-forward NN has been considered, having, besides the input layer, a hidden layer of neurons with tan-sigmoid transfer functions and an output layer of neurons with linear transfer functions. The training process is able to produce a network that minimizes the mean square error between the output **v**, which in this case coincides with the estimate **Θ̂**, and the actual value of the target parameter in the training dataset, that is the following quantity:

(12) $$\delta (\omega )=\sum_{h=1}^{H}{\left[\Theta (h)-\hat{\Theta }(h)\right]}^{T}[\Theta (h)-\hat{\Theta }(h)]$$

where *h* = 1:*H* denotes the *h*-th record of the training set. During training, the weights and biases are iteratively adjusted to minimize *δ*(**ω**). The minimization with respect to **ω** is usually based on repeated evaluation of the gradient of *δ*(**ω**) using the backpropagation algorithm. However, instead of the standard backpropagation, for a faster training, we used the Levenberg-Marquardt algorithm [45]. It is worth pointing out that the training process has been performed by monitoring the error between the network outputs and the targets on a test set independent from the training one, and stopping the process when a minimum of such error was found.

Note that the NN does not account explicitly for prior information about soil parameter statistics. However, as the training set reflects such properties of the quantities to be retrieved, the NN should be able to extract this information from it. Finally, it is worth underlining that the NN basically implements a MV criterion without any assumption on the statistics of the data.

## 4. Results

### 4.1. Results based on simulated data

**Figure 2.**
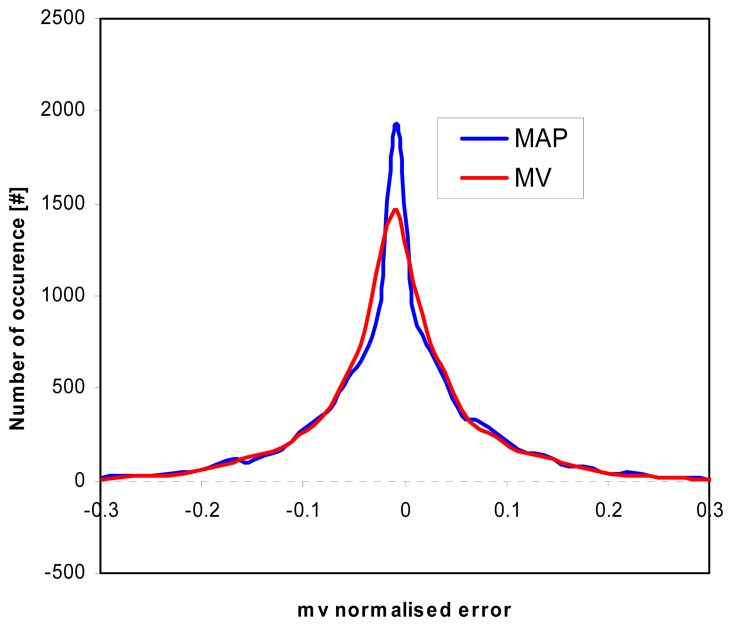
Comparison between histograms of the *m_v_* retrieval error obtained using MV and MAP (SEM derived database).

In this section we present the retrieval results using a simulated test set produced using both the IEM and the SEM direct models to simulate P (0.45 GHz), L (1.2 GHz), C (5.3 GHz), and X (9.66 GHz) band radar data. The accuracy of the estimation may be calculated in different ways. Here we have chosen to represent it in terms of the root mean square (rms) of the estimation error (the latter defined as the difference between the estimated and true values of the parameter to be retrieved), normalized to the prior standard deviation of the parameter, i.e. the standard deviation in the test database (hereafter normalized rms error). Evaluating the performances of the retrieval algorithms in terms of rms error, although fairly common, implicitly makes the MV- and NN-based estimators advantaged with respect to the MAP-based one, since the latter does not minimize the mean square error. It is interesting to compare the histograms of the error to analyze the effect of using different criteria. Figure 2 shows the histograms of the *m_v_* retrieval normalized error obtained by performing the inversion on a SEM derived database using MV and MAP (L, C and X bands). It can be noted that, although MV exhibits a smaller rms error, the number of occurrences of errors close to zero is larger when adopting MAP, which produces a more peaked histogram.

Different numbers of looks are considered when dealing with speckle effects. When *n* = 100, we intend that the simulations are carried out by considering radar data averaged within a homogeneous field made of several pixels. In other words, the 100-look exercise corresponds, in real conditions, to accomplish a “per field” analysis, assuming that a segmentation method has identified the single fields in the image, which have been averaged before carrying out the retrieval.

Tables 1 and 2 report the results of a comparison between the various algorithms. The test set has been generated by using SEM model for incidence angle *ψ*=20° (which allows achieving the best retrieval results for soil moisture in the absence of calibration errors, as shown later on), number of looks *n* = 100, L, C and X frequency bands (multifrequency analysis). Two situations are distinguished. In Table 1, no correlation is supposed between *s* and *l*, while, in Table 2, a correlation is established. Table 1 suggests that NN yields the best performances for all the soil parameters. However, if *s* is assumed to be correlated to *l*, when simulating the data and performing the retrieval, MAP and MV become comparable or even better than NN (Table 2). The normalized rms errors in Table 2 are smaller than those reported in Table 1, as expected, but the improvement is fairly small for NN if *m_v_* and *s* are considered. A priori information has therefore a considerable impact on the Bayesian methods (both MAP and MV). MV yields less normalized rms error with respect to MAP, since the latter does not minimize rms error, as previously pointed out. Fairly good performances are generally obtained for *m_v_* and *s*, while the low sensitivity (even if not absolutely zero) to *l* of the SEM-based simulations implies a large retrieval error for this parameter (almost 100% of the prior standard deviation).

**Figure 3.**
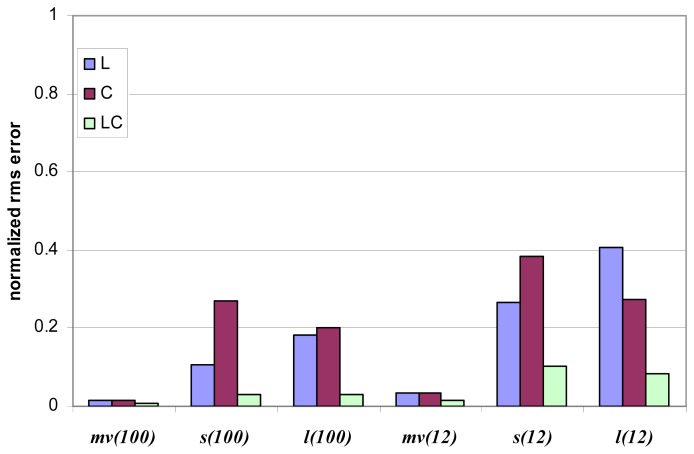
Accuracy estimation (in terms of normalized rms error) vs. frequencies and number of looks (reported between brackets) for IEM simulated measurements at *ψ*=20°. NN inversion method is applied.

**Figure 4.**
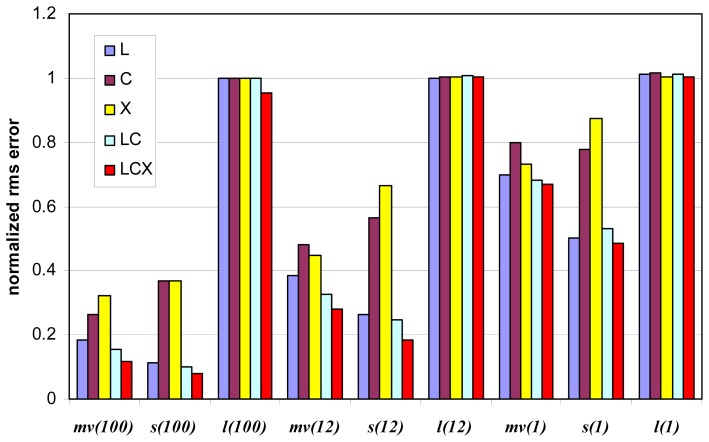
Same as Figure 3, but the simulations have been performed with SEM model and X band is also considered (both alone and in conjunction with L and C bands).

Figures 3 and 4 show an overview of the influence of number of looks and of frequency bands on the retrieval. As for Figure 3, IEM model has been used to produce the simulation, *n* is equal to either 12 or 100, L and C bands are considered (both single and multifrequency analysis). Figure 4 concerns SEM model, the number of looks is 1, 12 and 100 and the bands combination is L, C, X (single frequency), LC, and LCX (multifrequency analysis). NN is adopted and *ψ*=20°. It is possible to observe that the IEM model predicts a higher sensitivity of the radar measurements to the soil parameters, that is the predicted estimation accuracy is generally better compared to SEM. This is particularly true for *l*, as previously underlined, but also for *m_v_*, whereas for *s* the difference is less evident. It is understood that the differences, also in terms of frequency comparison, are related to the different sensitivity of the two models to the soil parameters. Anyhow, according to both models L band yields the best results for single band data. Acceptable results are obtained only through multilook summation. In this case, the multifrequency data compete with the signal fluctuation in a more effective way and improve the accuracy significantly.

**Figure 5.**
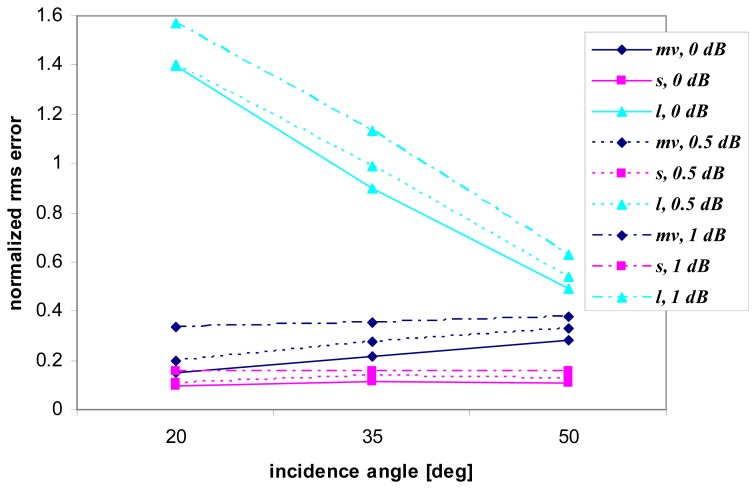
Accuracy estimation (in terms of normalized rms error) vs. incidence angle for SEM simulation, multifrequency (LCX) and multilook (*n*=100) analysis, MAP inversion method. An additive maximum error of 0.5 (dashed lines) and 1 dB (dotted lines) on the terms of the covariance matrix is considered. The case of no additive error is also included (solid lines).

To analyze the impact of other sources of discrepancies between **C** and **Z** matrices besides speckle, a systematic additive error has been introduced on the simulated database, which may account for model and instrument errors. The error has been uniformly generated with maximum values of 0.5 dB and 1 dB and independent values have been systematically applied to the three backscattering coefficients *σ^0^_hh_*, *σ^0^_vv_*, *σ^0^_vh_* and consequently computed for the other non-zero terms of the covariance matrix **C**, specifically <s_hh_s_vv_*> and its conjugate <s_vv_s_hh_*> Several datasets have been generated for testing the retrievals, with different sets of additive errors applied to the three backscattering coefficients. The effect on the estimation accuracy as a function of the incidence angle is shown in Figure 5 which concerns the MAP criterion, a test set built by means of the SEM model, multifrequency (LCX combination) and multilook (*n* = 100) analysis.

As expected, the accuracy generally decreases with the increase of the additive error. Figure 5 suggests that, although steeper incidences are more suitable for soil moisture in the absence of systematic errors, because of the reduced effect of roughness, *m_v_* retrievals at steeper angles are more sensitive to possible poor calibration of the data with respect to estimates accomplished at larger angles. Moreover we have found that, using the IEM simulated database, the rms error at lower incidence angles even overcomes that at more grazing angles. Basically, where sensitivity to the target parameter is higher, the degradation due to calibration errors is more relevant, so that intermediate angles can be preferable in the presence of poorly calibrated data.

### 4.2. Results based on experimental data

The model outputs have been compared to the polarimetric data acquired by the AIRSAR sensor (developed by JPL/NASA) during the MAC Europe Campaign in 1991 [46] and to the SIR-C/X-SAR data from Space Shuttle (1994) [47], over the test site of Montespertoli, Italy. Three frequency bands (P, L, C) were available from AIRSAR and two bands (L, C) from SIR-C, plus an X band at single polarization. Some fields of bare soil have been selected in the images whose soil moisture and roughness were measured during SAR acquisitions [48]. Soil moisture was determined by gathering soil samples with a probe of known volume, weighing them with a portable scale and drying them in a microwave oven. By using wet (*M*) and dry (*M*_2_) soil masses and the volume (*V*), the volumetric soil moisture values was calculated as [(*M*_1_−*M*_2_)/*V*]×100. The roughness of the selected fields was determined by different rural tillage (ploughed, harrowed, and rolled fields). The ranges of *s* and *l* for bare soils were approximately 1-5.5 cm and 3-19 cm, respectively [47]. Simulated against measured values of backscattering coefficients for L and C frequencies for the two campaigns have been compared.

**Figure 6.**
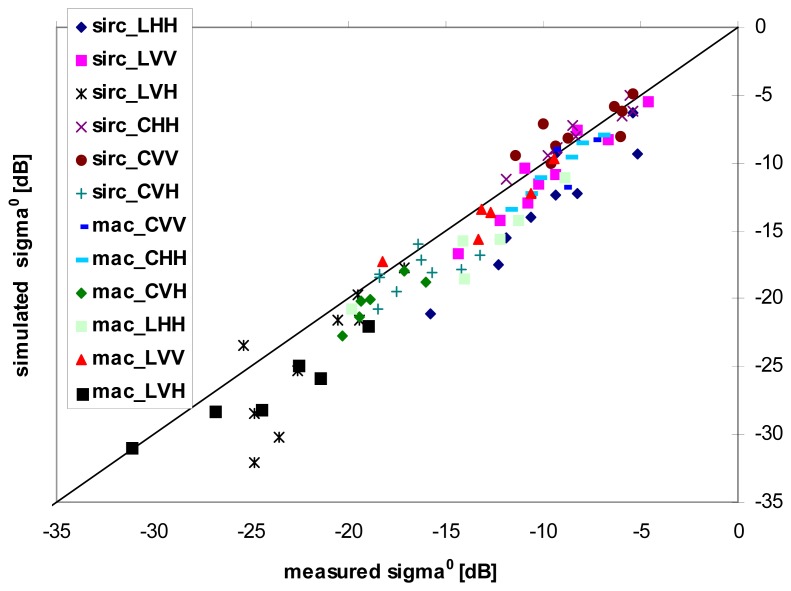
Scatterplot of SEM simulated versus measured backscattering coefficient (*σ*°) for MAC and SIR-C polarimetric data. Data are reported without any bias removal.

The SEM simulation provides a good matching with the values of *σ*° averaged within each field, as shown in Figure 6. However, a slight bias can be observed for MAC Europe data, in the order of 1-2 dB, depending on frequency/polarization channels. As for the IEM model, although it exhibits a better sensitivity to the soil parameters, as previously underlined (so that the expected retrieval performances were better when considering synthetic data), the results of the comparison between IEM-based simulations and actual backscattering coefficients have revealed less satisfactory. For this reason, in the following we refer only to the SEM model.

The bias between SEM simulations and real *σ^0^*, that is the mean difference between measured and simulated data at a given polarization, has been removed before applying the retrieval algorithm. In other words a “calibration” has been performed based on the simulations since a calibration error cannot be accounted for by the retrieval algorithm.

**Figure 7.**
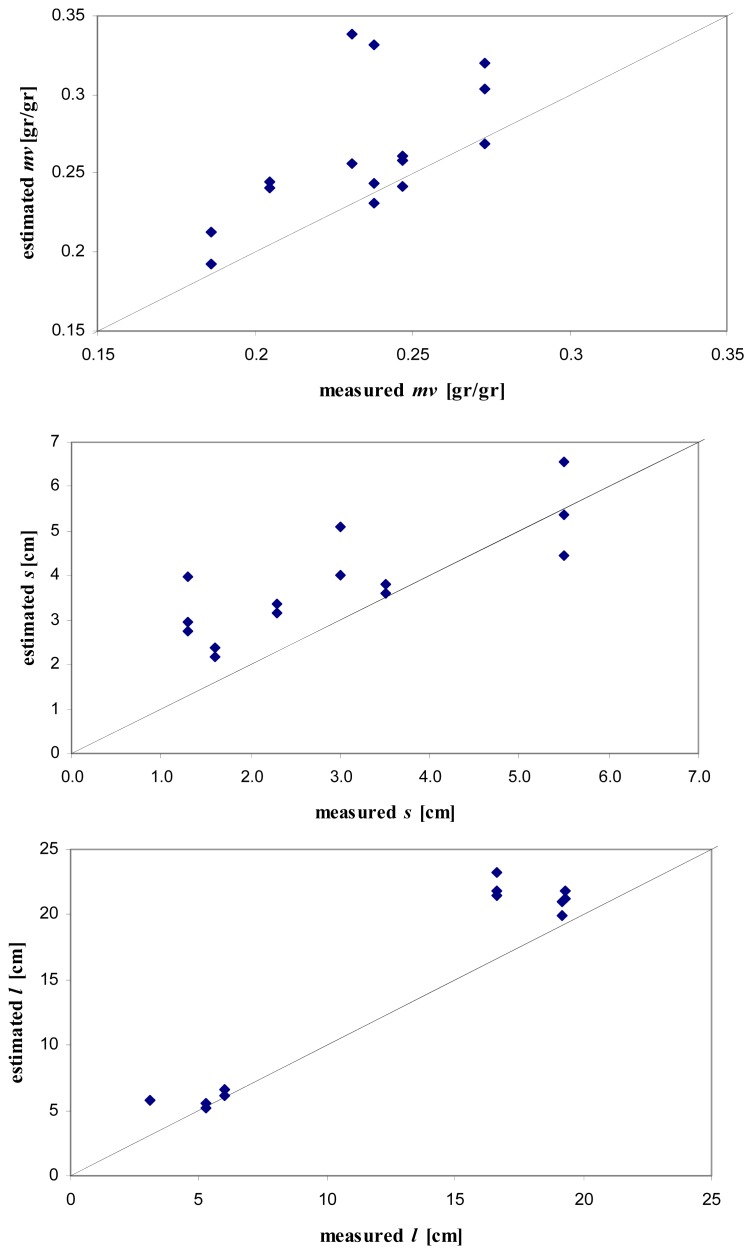
MV estimated versus measured bare soil parameters for MAC and SIR-C campaigns. Upper, central and lower panel concern soil moisture, standard deviation of height and correlation length, respectively.

The results obtained by using the MV criterion with the Monte Carlo integration are presented in Figure 7. The rms error is 1.3 cm for *s*, 0.044 for *m_v_* and a little overestimation of *s* is observed of about 0.9 cm. An error of 1.3 cm for *s* in the order of 3-4 cm means a relative error of about 30-40 %, whilst the simulations were predicting an error in the order of 20% for the same parameter. The error for *m_v_* has order of magnitude comparable to that expected from the simulation exercise reported in Table 2 (for non correlated case). The results for *s* show that some other uncertainties have affected the measurements, besides those predicted by the model. As for *l*, despite of the small sensitivity of SEM to this parameter, a rms error of 3.54 cm can be considered fairly encouraging, especially taking into account that the range of measurements of *l* is quite wide (between approximately 3 and 20 cm). We did not assume any correlation between *s* and *l* in the retrieval, and likely the algorithms achieved better results for *l* at the expense of the accuracy on *s*.

## 5. Conclusions

Different approaches for estimating bare soil parameters from multifrequency and polarimetric radar data have been described, to assess the potentiality of SAR polarimetry. They invert electromagnetic rough surface models in the framework of Bayesian criteria and Neural Network approaches. The methods have been tested on simulated data to compare their performances as function of number of looks, incidence angles and frequency bands. Neural Networks provide the best results when retrieval performances are measured by rms error (normalized to parameter standard deviation). MAP and MV criteria yield less accurate results, even though their clear theoretical framework and relatively small computer load can be appealing in some cases. By introducing *a priori* information regarding a correlation between *s* and *l*, the estimation of the parameters is improved and MAP and MV methods become comparable or even better than NN in terms of the normalized rms accuracy.

The analysis has also provided quantitative answers to some questions regarding the most profitable frequency band and incidence angle for accurate soil parameter retrieval. The L band provides best results when compared with other frequency bands. Lower incidence angles are better for *s* and *m_v_* estimation, but the accuracy is strongly reduced, mostly at lower angles, when a model/calibration error is considered. An acceptable accuracy for the parameter estimation is obtained only by multifrequency and multilook “per field” analysis.

The application of the retrieval methods to a small set of real data shows results which are comparable to the predicted one for soil moisture and less satisfactory for roughness standard deviation. A small bias between radar data and SEM simulations had to be removed. The accuracy obtained for *s* demonstrates that some unknown effects on the radar data can degrade the retrieval with respect to what was expected by the simulation exercise. As far as algorithms are concerned, the Monte Carlo approach for minimization/integration has the advantage to require running the forward model on a large set of soil samples only once, thus speeding up the retrieval.

This paper is intended to yield a contribution to find a reliable approach for bare soil parameter estimation from radar data, even though an ultimate solution to such a challenging problem is far to be obtained.

## Figures and Tables

**Table 1.** Comparison of retrieval results (in terms of normalized rms error) using MAP, MV and NN algorithms tested on a SEM-derived simulated database. Simulated data concern *n*=100, *ψ*=20°, L, C and X bands. No correlation is supposed between *s* and *l*.

**MAP**			**MV**			**NN**		
***m_v_*** [gr/gr]	***s*** [cm/cm]	***l*** [cm/cm]	***m_v_*** [gr/gr]	***s*** [cm/cm]	***l*** [cm/cm]	***m_v_*** [gr/gr]	***s*** [cm/cm]	***l*** [cm/cm]
0.178	0.128	1.377	0.198	0.097	1.083	0.142	0.099	0.987

**Table 2.** Same as Table 1, but assuming a statistical correlation between *s* and *l*.

**MAP**			**MV**			**NN**		
***m****_v_* [gr/gr]	***s*** [cm/cm]	***l*** [cm/cm]	***m****_v_* [gr/gr]	***s*** [cm/cm]	***l*** [cm/cm]	***m****_v_* [gr/gr]	***s*** [cm/cm]	***l*** [cm/cm]
0.114	0.086	0.561	0.101	0.082	0.397	0.138	0.096	0.516
